# The Tolerogenic Influence of Dexamethasone on Dendritic Cells Is Accompanied by the Induction of Efferocytosis, Promoted by MERTK

**DOI:** 10.3390/ijms242115903

**Published:** 2023-11-02

**Authors:** Vivien Li, Michele D. Binder, Trevor J. Kilpatrick

**Affiliations:** 1 The Florey Institute of Neuroscience and Mental Health, University of Melbourne, Parkville, VIC 3010, Australia; mbinder@florey.edu.au (M.D.B.); tkilpat@unimelb.edu.au (T.J.K.); 2 Department of Anatomy and Physiology, University of Melbourne, Parkville, VIC 3010, Australia

**Keywords:** dendritic cells, immune tolerance, efferocytosis, MERTK, dexamethasone

## Abstract

Many treatments for autoimmune diseases, caused by the loss of immune self-tolerance, are broadly immunosuppressive. Dendritic cells (DCs) can be induced to develop anti-inflammatory/tolerogenic properties to suppress aberrant self-directed immunity by promoting immune tolerance in an antigen-specific manner. Dexamethasone can generate tolerogenic DCs and upregulates MERTK expression. As MERTK can inhibit inflammation, we investigated whether dexamethasone’s tolerogenic effects are mediated via MERTK, potentially providing a novel therapeutic approach. Monocyte-derived DCs were treated with dexamethasone, and with and without MERTK ligands or MERTK inhibitors. Flow cytometry was used to assess effects of MERTK modulation on co-stimulatory molecule expression, efferocytosis, cytokine secretion and T cell proliferation. The influence on expression of Rab17, which coordinates the diversion of efferocytosed material away from cell surface presentation, was assessed. Dexamethasone-treated DCs had upregulated MERTK expression, decreased expression of co-stimulatory molecules, maturation and proliferation of co-cultured T cells and increased uptake of myelin debris. MERTK ligands did not potentiate these properties, whilst specific MERTK inhibition only reversed dexamethasone’s effect on myelin uptake. Cells undergoing efferocytosis had higher Rab17 expression. Dexamethasone-enhanced efferocytosis in DCs is MERTK-dependent and could exert its tolerogenic effects by increasing Rab17 expression to prevent the presentation of efferocytosed material on the cell surface to activate adaptive immune responses.

## 1. Introduction

Autoimmune disorders result from a loss of immune self-tolerance with the development of autoreactive T cells and auto-antibodies [1]. Current treatments for conditions such as multiple sclerosis and rheumatoid arthritis often involve therapies with broad immunosuppressive effects. However, these can increase the risk of infections and diminish vaccine responsiveness [2]. A more selective treatment approach is to suppress aberrant self-directed immune responses whilst promoting durable, stable immune tolerance in an antigen-specific manner, leaving general immune effector functions intact [1]. One method of achieving this is by harnessing dendritic cells (DCs), which are professional antigen presenting cells (APCs) and an essential bridge between the innate and adaptive immune systems. Treating DCs with anti-inflammatory signals can induce them to become tolerogenic, characterised by reduced maturation, downregulation of cell-surface major histocompatibility complex (MHC) and co-stimulatory molecules (CD40, CD80 and CD86), decreased pro-inflammatory and increased anti-inflammatory cytokine production and reduced capacity to activate adaptive immune responses [3]. Tolerogenic DCs (TolDCs) have been investigated as a therapeutic approach to autoimmune conditions, including multiple sclerosis [4,5], rheumatoid arthritis [6,7], type 1 diabetes [8] and Crohn’s disease [9].

Signalling mediated through the TAM (Tyro3, Axl, Mertk) receptors plays important roles in the immune system, including inhibiting the inflammatory response to pathogens and promoting the clearance of apoptotic cells by APCs. GAS6 is a ligand for all three receptors, whilst PROS can only activate TYRO3 and MERTK [10,11]. The TAMs have been explored as potential therapeutic targets in various contexts, including autoimmune disorders and cancer. They are expressed on cells of the immune (including DCs, macrophages, NK cells and B cells), nervous (microglia and astrocytes), reproductive and vascular systems [12].

Different compounds have been used to generate tolDCs in vitro, including dexamethasone, which upregulates MERTK expression at the mRNA and protein levels [13]. Dexamethasone exerts its tolerogenic effects through multiple pathways including the regulation of genes involved in DC maturation, complement activation and immune-related chemotaxis [14,15]. However, to what extent the diverse tolerogenic effects of dexamethasone are mediated via MERTK is not clearly defined. A previous study found that DCs with dexamethasone-induced MERTK upregulation could suppress human T-cell activation and proliferation. When DCs were treated with a blocking monoclonal antibody against MERTK, and subsequently co-cultured with CD4+ T-cells, T-cell proliferation and IFN-γ production increased [13]. However, the effects on CD4+ T-cell proliferation are only one of many amongst dexamethasone’s tolerogenic effects.

TAM receptors promote immune tolerance through several mechanisms [16] that may be potentiated for therapeutic purposes. TAM receptor signalling inhibits the production of pro-inflammatory cytokines, including TNF, IL-6, IL-12 and type I interferons in response to inflammation and Toll-like receptor (TLR) signalling pathways activated by pathogens [12]. Mice deficient in all three TAM receptors develop autoimmune conditions due to hyperproliferation of B- and T-cells, predicated on the loss of TAM receptors in APCs such as macrophages and DCs [17].

The TAM receptors also play a crucial role in modifying phagocytic uptake by APCs, serving to limit autoimmunity by clearing apoptotic cell debris that could otherwise serve as a source of autoantigens in a process known as efferocytosis. Efferocytosis is initiated when phosphatidylserine expressed by apoptotic cells is bound by PROS or GAS6, flagging these cells for APC engagement via bridging to one or more of the TAM receptors [18]. In APCs, the small GTPase recycling regulator Rab17 prevents efferocytosed material from loading onto MHC class II molecules, cell surface presentation and subsequent adaptive immune activation [19]. Animal studies have also demonstrated the key role of Mertk in efferocytosis and autoimmunity. Mertk-deficient mice have impaired clearance of apoptotic thymocytes by macrophages [20] and develop lupus-like symptoms [21]. Mertk-deficient non-obese diabetes mice develop disease earlier and at a higher frequency than their wild-type litter mates due to the failure of apoptotic cells to induce tolerance [22].

We aimed to interrogate the link between dexamethasone’s tolerogenic effects and MERTK signalling in DCs, as this could lead to strategies to potentiate these effects for therapeutic purposes by targeting MERTK signalling. We used two MERTK inhibitors, UNC2025 and MIPS15692, to block MERTK signalling on DCs. We hypothesised that, if treatment with the MERTK inhibitors could reverse the dexamethasone-induced changes in DC phenotypes, this would provide evidence for a substantive MERTK-mediated effect. Of the tolerogenic properties induced by dexamethasone, we found that only efferocytosis was MERTK mediated. We also provide a potential mechanism by which MERTK-dependent efferocytosis in DCs can contribute to immune tolerance.

## 2. Results

### 2.1. Monocyte-Derived Dendritic Cells Express Cell-Surface MERTK, Which Is Upregulated and Phosphorylated after Dexamethasone Treatment

Cells differentiated from CD14+ monocytes had good viability and appropriate phenotypic characteristics of monocyte-derived DCs (moDCs), including cell surface expression of HLA-DR, CD40, CD80, CD83, CD86 and DC-SIGN (a DC-specific marker [23]) (Supplementary Figure S1A), and appearance under light microscopy [24] (Supplementary Figure S1B).

**Figure 1:**
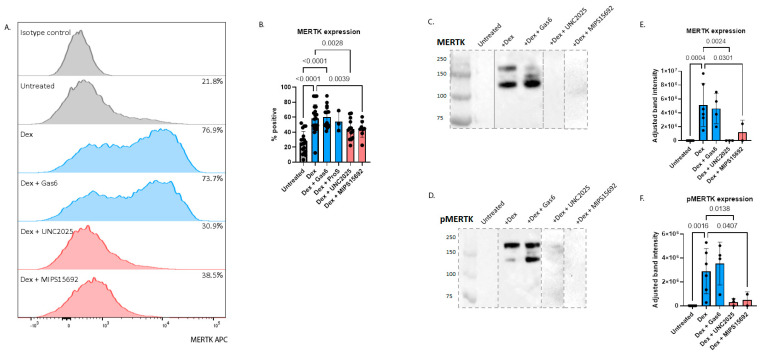
(**A**) Representative FACS histograms showing proportion of moDCs expressing surface MERTK in different treatment conditions. Proportions of moDCs expressing MERTK is increased by dexamethasone (*n* = 20) and decreased by either UNC2025 (*n* = 13) or MIPS15692 (*n* = 8) as assessed by (**B**) flow cytometry and (**C**) Western blot. (**C**,**D**) MERTK phosphorylation was also induced by dexamethasone (*n* = 6) and inhibited by UNC2025 (*n* = 3) or MIPS15692 (*n* = 2). Grey dotted lines indicate different blots that have been spliced together to remove extraneous lanes. (**E**,**F**) Level of MERTK expression and phosphorylation were quantified using densitometry. One-way ANOVA was used to compare means between groups followed by Fisher’s Least Significant Difference for pre-selected conditions indicated by comparisons shown in each figure.

To assess how MERTK signalling would affect tolerogenic properties in DCs, we firstly examined the expression of MERTK on moDCs under different conditions: untreated, with dexamethasone to upregulate MERTK, as well as with the MERTK ligands GAS6 and PROS and MERTK inhibitors UNC2025 and MIPS15692. Similar to previous studies [13], dexamethasone (10^−7^ M) treatment for 24 h upregulated MERTK on DCs (24.8% of untreated cells expressed surface MERTK versus 58.4% with dexamethasone [*p* < 0.0001]) (Figure 1A–C,E). Dexamethasone also induced phosphorylation of MERTK, as demonstrated by Western blot (Figure 1D,F). For both MERTK and phosphorylated MERTK (pMERTK), three bands of different molecular weights, likely representing differential glycosylation, were identified (Figure 1C,D).

We next assessed whether the addition of either GAS6 or PROS increased either MERTK expression or its phosphorylation in moDCs. The addition of GAS6 (100 ng/mL) to dexamethasone-treated moDC cultures for 24 h increased the intensity of pMERTK by approximately 1.8-fold (Figure 1F) but did not affect the proportions of cells expressing surface MERTK nor MERTK expression intensity (Figure 1A–C,E). Similarly, the addition of PROS (5 μg/mL) to dexamethasone-treated moDC cultures for 24 h did not alter the proportions of cells expressing surface MERTK (Figure 1A,B). Without dexamethasone, GAS6 did not significantly increase MERTK expression or induce phosphorylation (Supplementary Figure S2A,B).

The addition of either of the MERTK inhibitors UNC2025 (1 μM) or MIPS15692 (10 μM) to dexamethasone-treated moDC cultures for 24 h significantly reduced the proportions of cells expressing MERTK by approximately 25% (*p* = 0.0094 and *p* = 0.0291, respectively) (Figure 1A–C,E) and inhibited MERTK phosphorylation (Figure 1D,F). Taken together, these data indicate that dexamethasone had a predominant influence on inducing MERTK and pMERTK expression on moDCs, with either exogenous GAS6 or PROS having little added effect. On the other hand, both MERTK inhibitors significantly suppressed dexamethasone-induced MERTK expression and phosphorylation.

### 2.2. MERTK Signalling Mediates Dexamethasone-Induced Enhancement of Efferocytosis but Other Tolerogenic Effects Are MERTK-Independent

Next, we assessed the effect of dexamethasone on the following tolerogenic characteristics: expression of the co-stimulatory molecules CD40, CD80 and CD86, efferocytosis of myelin debris, cytokine secretion and T cell proliferation. Further, given the role of MERTK in immune tolerance and the upregulation of MERTK on moDCs with dexamethasone treatment, we also assessed whether any of the tolerogenic effects of dexamethasone are mediated directly via MERTK signalling by treating moDCs with the MERTK inhibitors UNC2025 (1 μM) or MIPS15692 (10 μM) for 24 h, in addition to dexamethasone.

**Figure 2:**
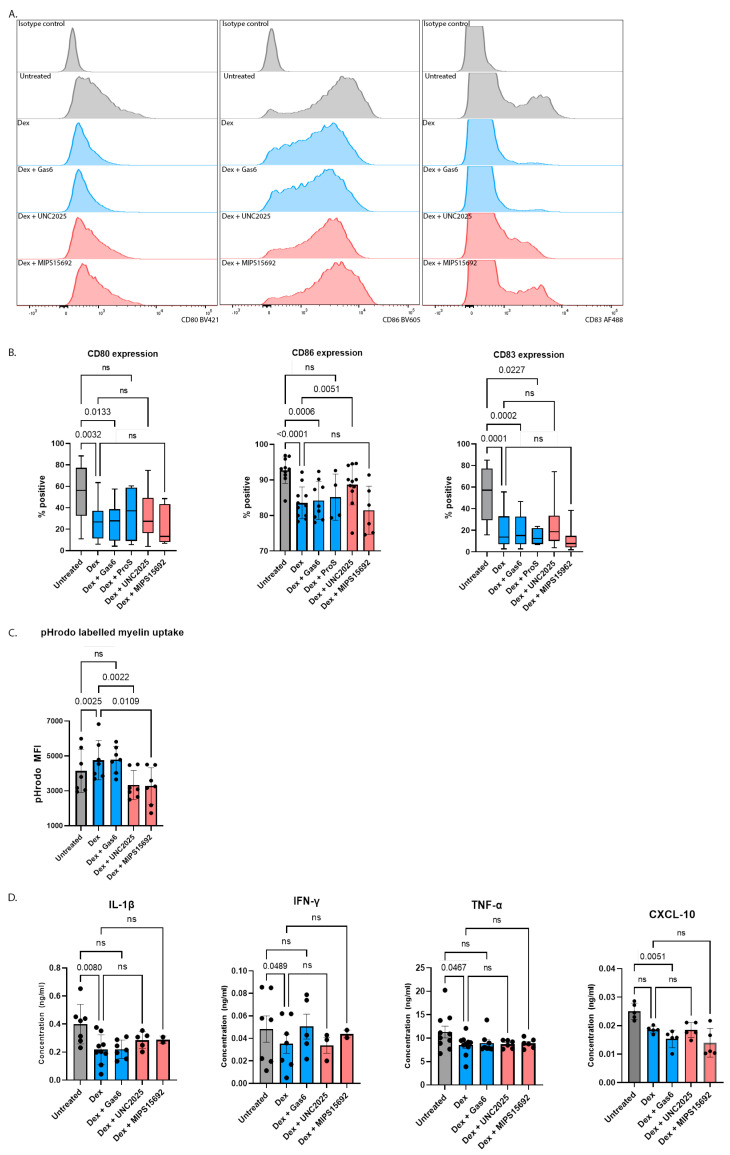
(**A**) Representative FACS histograms showing expression of cell surface CD80, CD86 and CD83 in different treatment conditions. (**B**) Dexamethasone (10^−7^ M) treatment for 24 h led to a lower proportion of moDCs expressing co-stimulatory molecules CD80 (*n* = 19) and CD86 (*n* = 10) as well as the mature DC marker CD83 (*n* = 21). UNC2025 (1 μM) and MIPS15692 (10 μM) for 24 h had discordant effects on expression of these cell surface markers. (**C**) Dexamethasone enhanced moDC uptake of pHrodo-labelled myelin debris, which was suppressed by both UNC2025 and MIPS15692 (*n* = 7). (**D**) Secretion of pro-inflammatory cytokines IL-1β (*n* = 7), IFN-y (*n* = 7) and TNF-α (*n* = 10) and chemokine CXCL-10 (*n* = 5) was suppressed in dexamethasone-treated moDCs cultures. Neither UNC2025 nor MIPS15692 had additional effect on cytokine production. One-way ANOVA was used to compare means between groups followed by Fisher’s Least Significant Difference for pre-selected conditions indicated by comparisons shown in each figure, with the exception of (**B**) CD80 and CD83 where Kruskal–Wallis test was used to compare means between groups followed by Dunn’s post-hoc test for pre-selected conditions indicated by comparisons shown in each figure. “ns” indicates the result was not statistically significant.

Compared to untreated moDCs, treatment with dexamethasone for 24 h resulted in significantly lower proportions of cells expressing the co-stimulatory molecules CD80 (54.2% versus 26.3%, *p* < 0.0001) and CD86 (92.7% versus 83.6%, *p* < 0.0001) (Figure 2A,B). The expression of CD40 was also reduced with dexamethasone but did not reach statistical significance (Supplementary Figure S3A). On the other hand, proportion of moDCs expressing HLA-DR were similar between the untreated and dexamethasone-treated groups (Supplementary Figure S3B).

Dexamethasone also reduced the proportions of cells expressing the mature DC marker CD83 (55% versus 20.3%, mean difference 34.7% [95% CI 27–42.4%], *p* < 0.0001), suggesting DCs were retained in a more immature state (Figure 2A,B). The expression of CD80, CD86 and CD83 were not further downregulated by co-stimulation of dexamethasone-treated moDCs with either GAS6 or PROS (Figure 2A,B) but CD40 expression was reduced further when PROS was added to dexamethasone (Supplementary Figure S3A).

We next assessed the influence of MERTK inhibition upon dexamethasone’s suppressive effects on CD80, CD86 and CD83 expression, comparing and contrasting the effects of UNC2025 (non-specific MERTK inhibition) [25] and MIPS15692 (specific MERTK inhibition) [26]. The addition of UNC2025 to dexamethasone for 24 h increased the proportions of CD83-expressing cells, indicating it could partly overcome the dexamethasone-mediated block of moDC maturation (Figure 2A,B). Similarly, expression of the co-stimulatory molecules CD80 and CD86, which were also suppressed with dexamethasone, increased when UNC2025 was added (Figure 2A,B). However, the addition of MIPS15692 produced no such effect; this is significant given that this kinase inhibitor has been shown to have greater specificity for MERTK than UNC2025 [25,26]. These data suggest that dexamethasone’s effects on CD80, CD86 and CD83 expression were not specifically mediated via MERTK signalling, but rather were orchestrated by the influence upon other receptor(s) inhibited by UNC2025.

Clearance of apoptotic cellular debris is an important anti-inflammatory mechanism. The influence of dexamethasone upon this process was assessed by incubating moDCs with pHrodo-labelled myelin debris for one hour subsequent to their exposure to dexamethasone for 24 h. Intracellular myelin debris could be detected using flow cytometry to assess the mean fluorescence intensity (MFI) of pHrodo green resulting from a pH change upon phagocytosis. Compared to untreated moDCs, dexamethasone led to an approximately 20% increase in pHrodo MFI (*p* = 0.0025) indicating increased uptake of myelin debris. The enhanced capacity to clear pro-inflammatory cellular debris is supportive of a tolerogenic phenotype (Figure 2C). The addition of GAS6 again did not further enhance myelin debris uptake. The addition of either UNC2025 or MIPS15692 had a concordant effect by completely reversing the dexamethasone-induced increase in engulfment of pHrodo-labelled myelin debris to below the level of untreated DCs (Figure 2C). Collectively, these data indicate that MERTK signalling is the predominant mechanism by which dexamethasone enhanced myelin debris uptake by moDCs.

Concentrations of cytokines and chemokines were measured in the DC culture supernatant. Dexamethasone treatment was associated with reductions in the pro-inflammatory cytokines IL-1β, IFN-γ and TNF-α, but the addition of GAS6, UNC2025 or MIPS15692 did not have any effect beyond that of dexamethasone alone (Figure 2D). The concentration of CXCL-10, an IFN-γ-dependent chemokine and Th1 chemoattractant [27], was reduced with dexamethasone with and without GAS6, but was unchanged with the addition of UNC2025 or MIPS15692 (Figure 2D). This again indicates that dexamethasone’s influence on cytokine and chemokine secretion was not MERTK-mediated.

**Figure 3:**
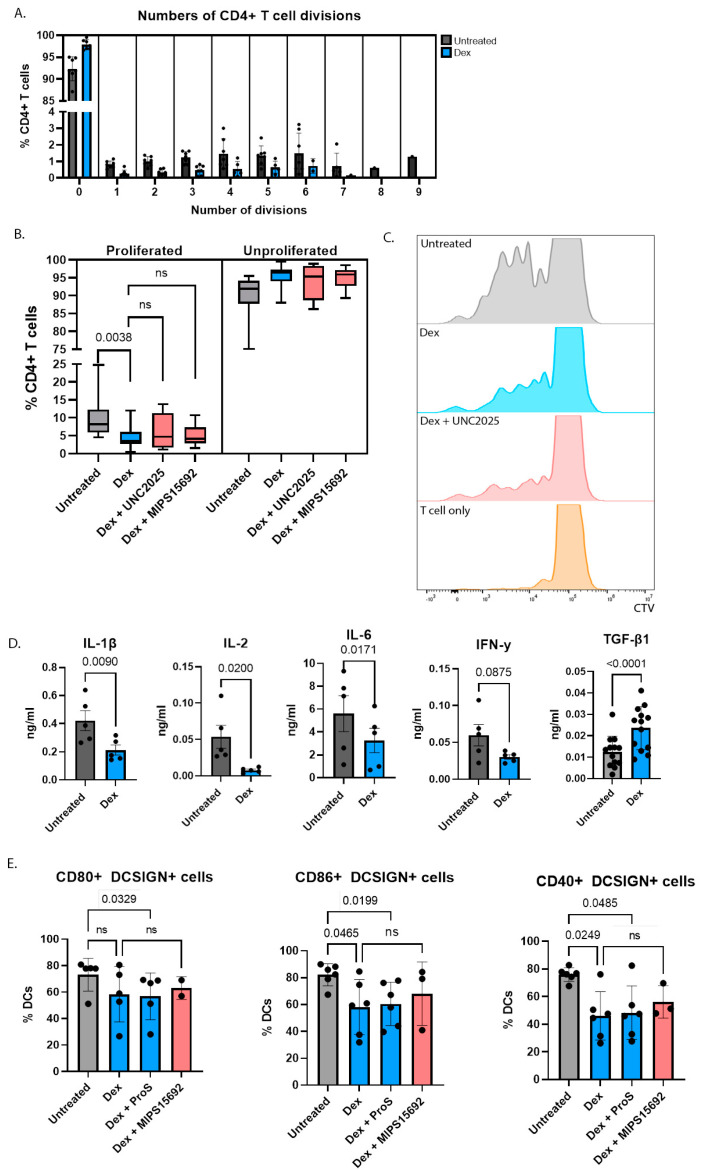
(**A**) Proliferation of CTV-stained CD4+ T-cell co-cultured with moDCs that had been pre-treated with dexamethasone (10^−7^ M) for 24 h was suppressed compared to when co-cultured with untreated moDCs (*n* = 16). The proportion of CD4+ T-cells that remained unproliferated (0 divisions) was higher when co-cultured with dexamethasone-treated moDCs, whereas CD4+ T-cells co-cultured with untreated DCs had undergone more divisions. (**B**) Treatment of DCs with neither UNC2025 (1 μM) (*n* = 8) nor MIPS15692 10 μM (*n* = 6) added to dexamethasone for 24 h had an additional effect on proliferation of co-cultured CD4+ T-cells. (**C**) Representative FACS histograms of CTV-stained CD4+ T cell proliferation when co-cultured with DCs that were untreated, treated with dexamethasone (10^−7^ M), dexamethasone (10^−7^ M) and UNC2025 (1 μM) for 24 h or in the absence of DCs. (**D**) Concentrations of pro-inflammatory cytokines (*n* = 5) were reduced and anti-inflammatory cytokine TGF-β (*n* = 13) was increased in dexamethasone-treated DC/T-cell co-cultures. (**E**) Proportions of DCs expressing CD80, CD86 and CD40 in DC/T-cell co-cultures were reduced with prior dexamethasone treatment but unchanged with addition of MIPS15692 (*n* = 5). *t*-tests or one-way ANOVA were used to compare means between groups followed by Fisher’s Least Significant Difference for pre-selected conditions indicated by comparisons shown in each figure, with the exception of (**B**) where Kruskal–Wallis test was used to compare means between groups followed by Dunn’s post-hoc test for pre-selected conditions indicated by comparisons shown in each figure. “ns” indicates the result was not statistically significant.

When activated, immunogenic DCs can stimulate proliferation of effector T-cells. Hence a key property of tolerogenic DCs is to suppress T-cell proliferation. We found that the proliferation of autologous CD4+ T cells was significantly reduced when they were co-cultured with dexamethasone-treated DCs compared to untreated DCs, as assessed by Cell Trace Violet dilution (Figure 3A–C). The effect of dexamethasone on suppressing proliferation was via the DCs rather than directly on T-cells, as dexamethasone was only present during initial DC differentiation, rather than during the subsequent period of DC/T-cell co-culture. Furthermore, CD4+ T cells cultured without DCs, either with or without dexamethasone, demonstrated minimal proliferation, with over 99% of cells remaining CTV-positive after five days (Figure 3C).

The mechanisms by which dexamethasone-treated DCs suppress CD4+ T cell proliferation are likely mediated via both the downregulation of co-stimulatory molecules on DCs, as well as the reduced secretion of pro-inflammatory cytokines. Concentrations of IL-1β, IFN-γ, TNF-α, IL-2 and IL-6 in supernatants from moDC culture and DC/T-cell co-culture were reduced with dexamethasone-treated compared to untreated DCs, whilst that of the anti-inflammatory cytokine TGF-β was increased (Figure 3D). In particular, IL-2 is a key cytokine involved in promoting T-cell proliferation, and its reduction in dexamethasone-treated DC/T-cell co-culture supernatant was associated with decreased proliferation.

Furthermore, the dexamethasone-induced reduction in the proportions of moDCs expressing the co-stimulatory molecules CD40, CD80 and CD86 persisted even after dexamethasone was removed from the culture media and autologous CD4+ T cells subsequently co-cultured with DCs for a further 5 days (Figure 3E), though the reduction in CD80 expression was no longer statistically significant. These data indicate that the tolerogenic effects of dexamethasone on suppressing T cell proliferation are enduring and mediated via an effect on moDCs rather than directly on CD4+ T cells.

The suppression of pro-inflammatory cytokines, as well as autologous CD4+ T cell proliferation in the presence of dexamethasone were unchanged by addition of either UNC2025 or MIPS15692, indicating these processes are MERTK-independent (Figure 2D and Figure 3B).

### 2.3. Rab17 Expression Was Increased in DCs Undergoing Efferocytosis

To further interrogate the mechanism by which dexamethasone-enhanced efferocytosis in DCs is tolerogenic or anti-inflammatory, we performed immunostaining for MHC-II and Rab17 in DCs cultured under different treatment conditions and then incubated with Cell Tracker Green-dyed apoptotic Jurkat cells to facilitate efferocytosis. Previous studies have shown that Rab17 mediates the transfer of apoptotic cellular debris to the recycling endosome away from the MHC-II compartment and subsequent cell surface presentation [19]. We hypothesised that there would be higher expression of Rab17 in cells undergoing efferocytosis, which would be further enhanced by dexamethasone and suppressed by addition of a MERTK inhibitor.

**Figure 4:**
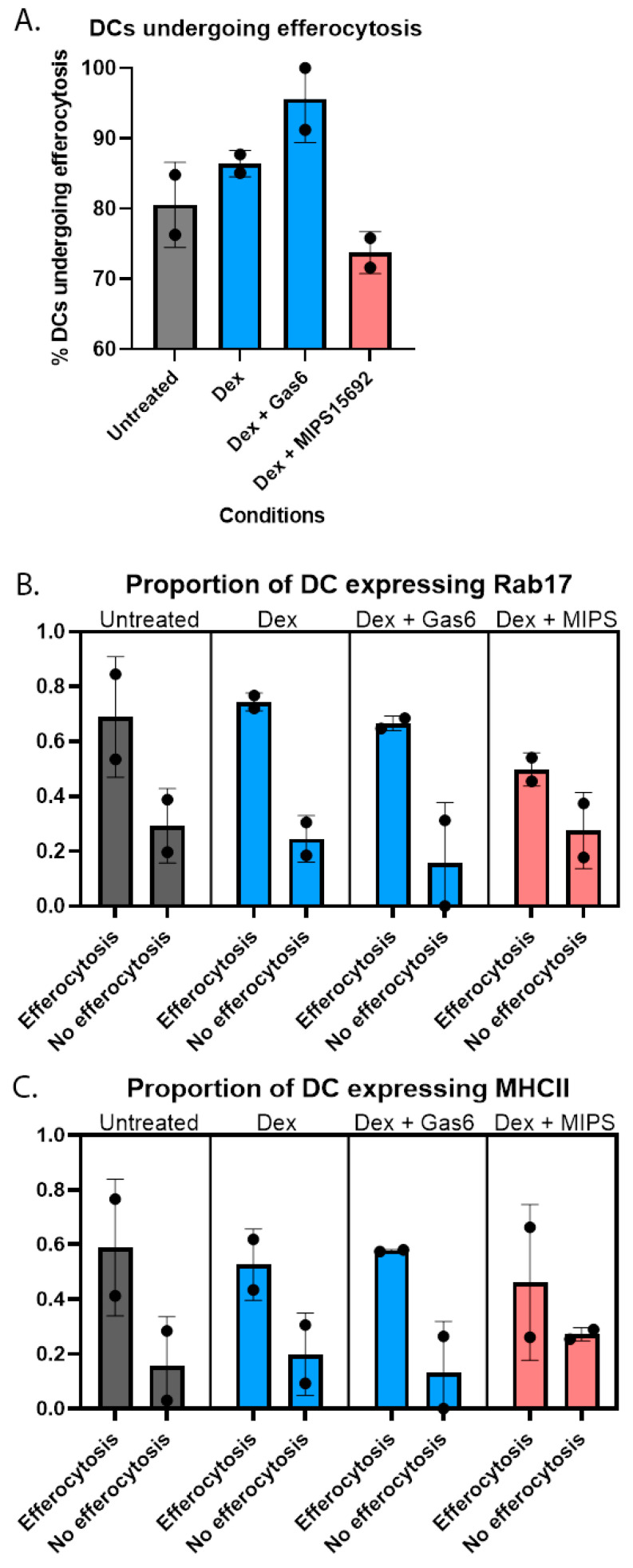
(**A**) Proportions of moDCs undergoing efferocytosis were higher in dexamethasone and dexamethasone + GAS6 conditions compared with untreated and dexamethasone + MIPS15692 conditions (*n* = 2). (**B**) A higher proportion of cells undergoing efferocytosis expressed Rab17 in all conditions compared to cells not undergoing efferocytosis with lower expression in cells treated with MIPS15692 (*n* = 2). (**C**) A higher proportion of cells undergoing efferocytosis expressed MHC-II in all conditions compared to cells not undergoing efferocytosis with no differences between conditions (*n* = 2).

We first confirmed that the proportions of DCs undergoing efferocytosis, quantified by numbers of nucleated cells bound to apoptotic Jurkat cells, was increased in the dexamethasone and dexamethasone + GAS6 conditions compared to untreated and dexamethasone + MIPS15692 conditions (Figure 4A).

In all treatment conditions, Rab17 was expressed in a higher proportion of DCs that were undergoing efferocytosis than those that were not. There was higher Rab17 expression in the dexamethasone and dexamethasone + GAS6 conditions compared to dexamethasone + MIPS15692 condition (Figure 4B). Proportions of DCs expressing MHC-II were higher in cells undergoing efferocytosis in all treatment conditions (Figure 4C). This latter observation is consistent with findings from flow cytometry of similar proportions of moDCs expressing HLA-DR (Supplementary Figure S3B). This finding supports the hypothesis that efferocytosis is also associated with higher DC expression of Rab17, a protein responsible for diverting antigen presentation away from cell surface MHC-II and that this effect was suppressed when MERTK signalling was inhibited by MIPS15692.

### 2.4. Dexamethasone-Induced Tolerogenic Characteristics Are Stable after Lipopolysaccharide Exposure

To develop tolerogenic DCs as a potential therapy to induce immune tolerance in autoimmune diseases, tolerogenic characteristics must remain stable in a pro-inflammatory environment when administered in vivo. To test for this enduring capacity, moDCs generated after 48 h of treatment with GM-CSF and IL-4 followed by a cocktail of maturation stimuli and dexamethasone added on day 1 were subsequently exposed to lipopolysaccharide (LPS) for 24 h after the dexamethasone had been removed. Furthermore, to assess whether the effects of the MERTK inhibitors on reversing the tolerogenic effects induced by dexamethasone were also sustained, moDCs that had been treated with dexamethasone and UNC2025 or MIPS15692 for 24 h were exposed to a further 24 h of LPS without dexamethasone or the MERTK inhibitors.

**Figure 5:**
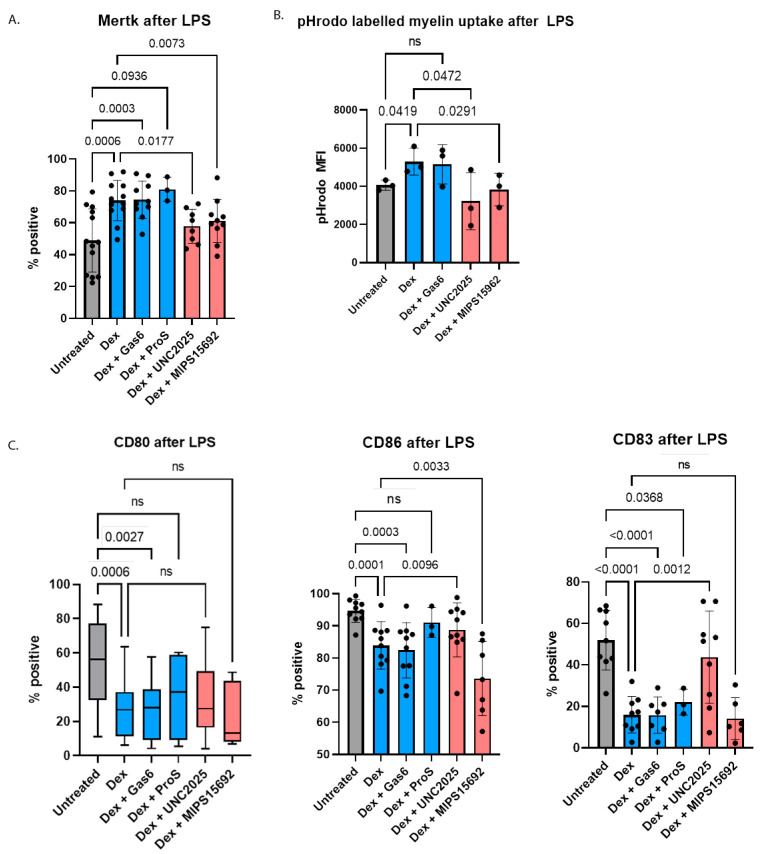
(**A**) Dexamethasone-induced upregulation of MERTK expression (*n* = 13), (**B**) enhancement of myelin debris uptake (*n* = 3) and (**C**) downregulation of CD80 (*n* = 19), CD86 (*n* = 10) and CD83 (*n* = 9) expression on moDCs was maintained after further exposure to LPS 100 ng/mL for 24 h in the absence of dexamethasone. One-way ANOVA was used to compare means between groups followed by Fisher’s Least Significant Difference for pre-selected conditions indicated by comparisons shown in each figure, with the exception of (**C**) CD80 where Kruskal–Wallis test was used to compare means between groups followed by Dunn’s post-hoc test for pre-selected conditions indicated by comparisons shown in each figure. “ns” indicates the result was not statistically significant.

Proportions of cells expressing surface MERTK remained significantly higher in the group previously treated with dexamethasone compared to untreated cells (74% versus 48.9%, *p* < 0.0001). This demonstrates that the effect of dexamethasone on upregulating MERTK expression was enduring, despite LPS exposure (Figure 5A). The proportions of MERTK-expressing cells remained lower in the dexamethasone plus UNC2025 or dexamethasone plus MIPS15692 conditions, compared to the dexamethasone only condition. However, these inhibitory effects were reduced after the MERTK inhibitors were removed from the culture media, independent of the presence or absence of LPS over the subsequent 24 h (Figure 5A).

The capacity of moDCs to take up myelin debris, which was enhanced by dexamethasone, persisted after subsequent exposure to LPS. The effects of prior MERTK inhibition with 24 h of UNC2025 or MIPS15692 on suppressing myelin debris uptake were also sustained with significant reductions in pHrodo MFI even after the MERTK inhibitors were subsequently removed (Figure 5B). The dexamethasone-induced reduction in cells expressing co-stimulatory molecules CD80 and CD86, as well as the mature DC marker CD83 also remained after LPS treatment for a further 24 h without dexamethasone being present, indicating cells were maintained in an immature and less pro-inflammatory state (Figure 5C).

## 3. Discussion

We have shown that dexamethasone significantly upregulates MERTK expression and induces MERTK phosphorylation in moDCs. Dexamethasone treatment also promotes a tolerogenic phenotype characterised by a combined reduction in co-stimulatory molecule expression, moDC maturation, capacity to stimulate proliferation of CD4+ T-cells and pro-inflammatory cytokine secretion, whilst enhancing efferocytosis of myelin debris. MERTK is known to be involved in immune tolerance and by using MERTK inhibitors we demonstrated that the tolerogenic effects of dexamethasone on efferocytosis are mediated via MERTK signalling, and this MERTK-mediated enhancement of efferocytosis could facilitate diversion of apoptotic materials towards recycling endosomes and away from MHC-II presentation. However, other dexamethasone-induced tolerogenic effects, including reducing co-stimulatory molecule expression, DC maturation, pro-inflammatory cytokine production and T-cell proliferation, appear to be MERTK-independent. The influences of dexamethasone upon MERTK expression and promotion of tolerogenic characteristics in moDCs were enduring despite subsequent exposure to a pro-inflammatory stimulus, namely LPS, in the absence of dexamethasone.

Tolerogenic DCs are emerging as a promising therapeutic approach to autoimmune diseases. Dexamethasone is one of several agents that can promote a tolerogenic phenotype in DCs in vitro. Dexamethasone has diverse effects but the mechanisms by which it induces these properties are not clearly defined. One of the unique effects of dexamethasone, compared to other tolerogenic agents such as vitamin D3 and TGF-β, is to upregulate MERTK expression on DCs [13,14]. Given the role of MERTK in immunomodulation, we sought to assess to what extent the tolerogenic phenotype induced by dexamethasone is mediated via MERTK signalling, as this could provide insights into how to harness this for therapeutic effect.

Firstly, we confirmed that the proportion of moDCs expressing MERTK increased by over twofold with dexamethasone treatment, which is consistent with previous studies [13]. MERTK upregulation was also associated with its phosphorylation, which is required for its activation and downstream actions [16]. Interestingly, on Western blot, in addition to detecting the expected dominant MERTK and pMERTK species at approximately 110 kDa and 180 kDa, we also identified two fainter bands, one at a higher and the other at a lower molecular weight, seen with dexamethasone treatment either with or without additional GAS6. These likely represent differentially glycosylated forms of MERTK [28]. The MERTK contains 14 N-linked glycosylation sites in its extracellular domain and several differentially glycosylated forms have been reported ranging from 110 kDa to 205 kDa [28]. There is evidence from tumour cell types that differentially glycosylated MERTK isoforms are functionally important in the context of MERTK’s role in oncogenesis. A previous study using acute lymphoblastic leukaemia cell lines found that prolonged GAS6 exposure for over 18 h led to preferential expression of a lower molecular weight partially glycosylated form of MERTK compared to control samples where the fully glycosylated form was predominant [29]. The partial MERTK glycoform was associated with altered downstream ERK signalling and localisation to nuclear compartments rather than the plasma membrane. MERTK also contains a nuclear localisation sequence, suggesting nuclear MERTK could be involved in transcriptional regulation following prolonged ligand stimulation [30]. MERTK N-glycosylation was also found to be critical for its homodimerisation, stability and tumour-promoting effect in hepatocellular carcinoma [31]. Whether the glycoforms of MERTK and pMERTK have different functions and localisation in DCs is unclear. We also found that prolonged GAS6 treatment, together with dexamethasone, was associated with a relative increase in the lower molecular weight form, suggestive of reduced glycosylation, although this would have to be verified via the addition of tunicamycin. Of note, a recent study identified MERTK in the nucleus of human monocyte-derived DCs, with translocation induced by the binding of protein S [32]. The highest nuclear localisation was found in newly differentiated DCs, but dexamethasone treatment led to only a minor increase in nuclear MERTK. Nuclear MERTK was associated with open chromatin and was proposed to act as a transcription factor regulating DC differentiation, though the authors were not able to identify the genomic regions influenced by MERTK.

The mechanisms by which dexamethasone exerts tolerogenic effects has been previously studied by transcriptomic profiling of DCs. Dexamethasone was associated with the downregulation of genes involved in DC maturation and inflammation, including CD80, CD83, CD1c, ACTA2, ACTG1, TMBS10, AP-1 and RAP1GAP, and the upregulation of anti-inflammatory genes, including MERTK and IL-10, involved in expansion of Treg cell populations and tolerance [14]. These changes in gene expression are in concord with our findings at a protein level with reduced CD80 and CD83 expression on flow cytometry. Another study performed microarray analysis of dexamethasone-treated DCs to identify the mechanisms of tolerance induction [15]. Overexpressed genes in dexamethasone-treated compared to untreated DCs included those involved in immune-related functions such as complement activation (C1QB and C1QC), immune-related chemotaxis (CCL2, CCL4, CCL18 and CCL26), CD163 and the induction of ERK1/2 signalling. In particular, C1Q has been implicated in tolerogenic processes such as the clearance of self and apoptotic cells by macrophages and DCs, by acting as a bridge between phagocytic cells and apoptotic debris [33]. C1Q-stimulated DCs also demonstrated reduced expression of CD80, CD83 and CD86, reduced capacity to stimulate T-cell proliferation and the production of pro-inflammatory cytokines [34], changes that were observed in our dexamethasone-treated DCs.

As MERTK is involved in immune regulation and is upregulated by dexamethasone on DCs, we sought to assess whether MERTK signalling is directly involved in the tolerogenic phenotype induced by dexamethasone by adding either a non-specific (UNC2025) or specific (MIPS15692) MERTK inhibitor to dexamethasone-treated moDCs. UNC2025 is a small-molecule tyrosine kinase inhibitor that potently inhibits MERTK (IC_50_ = 2.7 nM) and FLT3 (IC_50_ = 3 nM), along with multiple other receptors at higher concentrations. It is selective towards MERTK relative to other TAM receptors including Axl, the next potently inhibited kinase (IC_50_ = 122 nM) [25]. It has been used in previous studies investigating the role of MERTK in myelin phagocytosis by human myeloid cells [35]. MIPS15692 also has potent activity against MERTK (IC_50_ = 4.0 nM) with good selectivity for MERTK over AXL (∼40×) and TYRO3 (∼85–100×), and importantly, does not act on FLT3 [26]. Both agents abolished MERTK and pMERTK expression in DCs. They could fully reverse the dexamethasone-induced enhancement of myelin debris uptake by moDCs, indicating that this action is mediated via MERTK signalling. This is in keeping with a known role of MERTK in the clearance of apoptotic cell debris via efferocytosis. Phosphatidylserine expressed by apoptotic cells is bound by the TAM ligands, which flag them for TAM receptor-mediated efferocytic uptake by APCs [18].

The processes by which phagocytic cells such as DCs take up material can determine whether downstream immunogenic or tolerogenic processes are initiated. Phagocytosis of pathogens activates pro-inflammatory responses with antigen degradation and presentation on cell surface MHC-II. Conversely, following efferocytosis of apoptotic cells that would otherwise promote inflammation if left uncleared, the engulfed material is transferred to recycling endosomes, diverting it away from the MHC-II loading compartment, subsequent cell surface presentation and T-cell activation. In a previous study, the GTPase Rab17 was found to be induced in macrophages following efferocytosis and involved in this differential processing [19]. Rab17 is known to play a key role in regulating the recirculation of material in polarised cells via recycling endosomes, as demonstrated by its colocalization with the recycling endosome markers transferrin receptor and FcLR chimeric receptor [36]. In a study by Yin et al., Rab17 was recruited to both phagosomes and efferosomes in early stages after engulfment of the target material but persisted only in the latter. The authors proposed that initial Rab17 activation and recruitment was induced by the guanine exchange factor Rabex-5 [37], though other molecules such as Rab7 may subsequently displace Rabex-5 from phagosomes [19]. We also found a higher expression of Rab17 in DCs undergoing efferocytosis. Whilst this was the case in all treatment conditions, Rab17 expression was lower in DCs that had been treated with MIPS15692 compared to dexamethasone with or without GAS6, suggesting that MERTK inhibition correlated with reduced recycling of engulfed apoptotic material. Further replication is required to confirm these promising data, which point to modulation of MHC-II antigen presentation of apoptotic material by DCs and subsequent activation of adaptive immune responses as a potential mechanism linking dexamethasone-enhanced efferocytosis and MERTK signalling with its tolerogenic effects.

Previous studies have also linked apoptotic cells with inhibition of DC maturation and activation via the NF-κB pathway, a process found to be dependent on MERTK activation of the PI3K/AKT pathway in a non-obese diabetes mouse model [38]. The suppression of NF-κB transcription also inhibits secretion of pro-inflammatory cytokines, such as TNF-α, IL-1β, IL-6 and IL-12 [39,40]. The effect of apoptotic cells on DCs was found to be GAS6-dependent, and MERTK-deficient mice exhibited increased frequency of activated pancreatic DCs with enhanced T cell stimulatory capacity [22]. This suggests that MERTK is important for this process in vivo, whereas dexamethasone has similar effects in vitro but via alternative mechanisms.

We found that other tolerogenic effects of dexamethasone, including upon the inhibition of co-stimulatory molecule expression, maturation, pro-inflammatory cytokine production and T-cell proliferation, were not MERTK-mediated. Whilst UNC2025 could partly reverse the dexamethasone-induced inhibition of CD80 and CD86 expression and DC maturation, this effect was not seen when the specific MERTK inhibitor MIPS15692 was added to dexamethasone-treated moDCs. UNC2025 is a potent inhibitor of both MERTK and FLT3. In addition, it inhibits a number of other receptors at higher IC50 levels, including AXL, TRKA, TRKC, QIK, TYRO3, SLK, NuaK1, KIT and Met [25]. At the concentration of 1 μM used in most of our experiments, it would be expected that both MERTK and FLT3 were completely inhibited whilst the other receptors would also reach greater than 90% inhibition. FLT3 signalling is also required for DC differentiation. The administration of FLT3 ligand to both mice and human subjects resulted in the expansion of circulating DCs in vivo [41,42]. Whilst these DCs had low expression of CD40, CD80 and CD83, representing an immature state, CD80, CD83 and CD86 expression could be upregulated following further in vitro culture of Flt3L-mobilised DCs [43]. Given the discordant impact of non-specific and specific MERTK inhibition using UNC2025 and MIPS15692, respectively, on many of the dexamethasone-induced characteristics, it is tenable that FLT3 rather than MERTK signalling underlie these effects confined to UNC2025.

The addition of the MERTK ligands GAS6 and PROS did not enhance dexamethasone’s tolerogenic effects, further supporting that most of the changes were not MERTK-mediated. Interestingly however, GAS6 also did not enhance the efferocytic capacity of DCs. This may be due to sufficient GAS6 or PROS being present in the culture environment, such that adding exogenous ligand had no further effect. In support of this view, secreted GAS6 and PROS have been detected in serum-free cultures of mice peritoneal macrophages [44] and GAS6 from mice hepatic stellate cells [45].

The role of TAM signalling in the interaction between DCs and T cells is clearly complex. In contrast to our findings, a previous study that investigated the role of MERTK in immune tolerance using a neutralising antibody against MERTK rather than a small molecule inhibitor found that blockage of MERTK in DC-T cell cultures increased CD4+ T cell proliferation and IFN-γ production [13]. This study reported that the activation of MERTK signalling reduced the secretion of IL-2, involved in expansion of T-cell populations, which may underlie MERTK-mediated T-cell suppression [13]. Whilst we also observed inhibition of T-cell proliferation and reduced IL-2 secretion in dexamethasone-treated moDC/T-cell co-cultures, MERTK inhibition using UNC2025 or MIPS15692 did not reverse this effect, to increase CD4+ T-cell proliferation. Of note, there is also negative feedback from activated T-cells which can express PROS to limit DC activation via TAM signalling at the T cell–DC interface to maintain immune homeostasis. Neutralisation of PROS with an anti-PROS antibody was found to increase CD86 and CD40 expression on DCs [46]. It is therefore possible that, in certain contexts, inhibiting MERTK may also attenuate the suppressive effect of activated T cell-derived PROS on limiting further DC activation.

Discrepancies in outcomes have also been observed in studies assessing the effects of MERTK inhibition using different methods and models both in vivo and in vitro. A recent study found that phenotypic characteristics which have been traditionally attributed to the loss of MERTK in a widely used MERTK knockout mouse model established by Camenisch et al. cannot be explained by loss of MERTK function alone [47]. For example, retinal degeneration seen in these mice, which had been previously ascribed to failure of MERTK-mediated phagocytosis of photoreceptor outer segments, is only present when TYRO3 function is also lost and was not seen in independently generated MERTK knockout mice. On the other hand, a study focusing on the type 1 diabetes NOD mouse model, and assessing granzyme B and IFN-γ production as markers of CD8+ and CD4+ T-cell activation following MERTK inhibition with UNC2025 challenge, found incomplete penetrance. Only half of these challenged mice showed an increase in granzyme B expression and disease induction, whilst IFN-γ production was unaffected, indicating there are potentially other mechanisms of immune tolerance compensating for MERTK signalling [48].

In vitro studies assessing TAM receptor signalling have previously predominantly used either monoclonal antibodies or small molecule inhibitors. However, the former can potentially lead to receptor dimerization and internalisation, and the activation of kinases during internalisation [49], whilst compensatory upregulation of receptor expression has been observed with small molecule inhibitors targeting AXL [50]. A recent study demonstrated proof-of-concept of another method for TAM receptor inhibition using targeted protein degraders which could prove useful in mitigating against these confounding effects [51]. The methodology involved applying heterobifunctional molecules that use the ubiquitin proteasome pathway to target specific proteins for degradation by redirecting E3 ubiquitin ligase activity. The authors report that these molecules are not dependent on sustained target engagement to maintain the desired effect, but rather initiate an event driven process. A selective MERTK degrader reduced bone marrow-derived macrophage MERTK cell surface expression by up to 70%, and MERTK expression remained suppressed below baseline levels for at least 24 h after the removal of the degrader. Treatment also led to decreased efferocytosis of apoptotic Jurkat cells. This highlights the importance of interrogating functions of the TAM receptors using different models and methods and ultimately understanding the complex influence that any given intervention can have upon signalling mechanisms.

In conclusion, dexamethasone induces a number of tolerogenic characteristics in DCs. One of its unique effects compared to other tolerogenic agents is to upregulate the tyrosine kinase receptor MERTK and induce its phosphorylation. With the current interest in MERTK as a therapeutic target in autoimmune diseases, and indeed its use as a marker of tolDCs in human trials [5,9], it is important to understand the mechanisms by which MERTK signalling could promote a tolerogenic phenotype in DCs. By using non-specific and specific MERTK inhibitors, we were able to distinguish between MERTK-dependent and independent tolerogenic effects of dexamethasone and to propose a further mechanism by which efferocytosis-mediated immune tolerance is mediated. It is not unexpected that there are redundancies in the pathways underpinning the execution of a critical process such as immune tolerance. Our work provides further clarification of how MERTK activation can contribute to this process and potentially for optimised therapeutic benefit.

## 4. Materials and Methods

### 4.1. Peripheral Blood Mononuclear Cell Isolation

Buffy coat samples from the Australian Red Cross (obtained under Material Supply Deed 20-03VIC-02 and 22-04VIC-09) and whole blood samples from healthy controls (ethics approval from Melbourne Health Human Research Ethics Committee project number 2013.111) were used for peripheral blood mononuclear cell (PBMC) isolation. Samples were diluted 1:1 in room temperature phosphate buffered saline (PBS) (without calcium and magnesium) in 50 mL Falcon centrifugation tubes (Fisher Scientific, Waltham, MA, USA) and mixed gently using a serological pipette. Then, 30 mL of diluted samples was carefully added to another centrifugation tube containing 15 mL of room-temperature Ficoll-Paque Plus (GE Healthcare), avoiding mixing and introduction of air bubbles. Tubes containing Ficoll-Paque Plus and the diluted blood mixture were centrifuged at room temperature for 30 min at 700× *g* with medium acceleration and no brake (Eppendorf Centrifuge 5910R with swinging bucket rotor). The PBMC layer between the PBS/plasma and Ficoll layers was carefully collected and transferred into a new 50 mL centrifugation tube and PBS was added to increase the sample volume to 50 mL to dilute any Ficoll that may have been aspirated. Tubes were centrifuged for 15 min at 480× *g* with full brake. The supernatant was discarded and cell pellet loosened by tapping the base of the tube. All cell pellets from each individual were pooled into a single centrifugation tube and PBS was added to increase the sample volume to 10 mL. Then, 10 µL was diluted with 90 µL Trypan Blue Solution 0.4% in an Eppendorf microtube (ThermoFisher Scientific, Waltham, MA, USA) for counting, using a Neubauer haemocytometer. After counting, the 10 mL PBMC suspension was completed to 50 mL with PBS and centrifuged for 12 min at 310× *g* with full brake. The supernatant was discarded and cell pellet containing PBMCs used for subsequent CD14+ monocyte isolation.

### 4.2. CD14+ Monocyte Isolation

PBMCs were resuspended in 1 µL Fc receptor blocking antibody (130-059-901, Miltenyi Biotec, Bergisch Gladbach, Germany) diluted in 49 µL fluorescence-activated cell sorting (FACS) buffer (2% foetal calf serum, 2 mM EDTA and PBS) per 10^6^ cells for 10 min at 4 °C. FACS buffer was added to increase the sample volume to 5 mL before centrifuging at 300× *g* for 10 min at 4 °C to form a cell pellet. Cells were incubated with 20 μL CD14 magnetic microbeads (130-050-201, Miltenyi Biotec, Germany) diluted in 80 μL of FACS buffer per 10^7^ cells for 30 min at 4 °C. FACS buffer was added to increase the cell suspension volume to 2 mL before centrifuging at 300× *g* for 10 min at 4 °C to remove unbound microbeads. Cells were resuspended in 500 μL FACS buffer and loaded into a MACS column pre-rinsed with 500 μL of FACS buffer that was placed in the magnetic field of a MACS Separator (Miltenyi Biotec, Bergisch Gladbach, Germany). A maximum of 2 × 10^8^ cells was used per column. The MACS column was washed three times with 500 μL of FACS buffer, then removed from the separator and placed in a 15 mL collection tube. A further 1 mL of FACS buffer was used to flush out the magnetically labelled CD14+ cells.

### 4.3. Generation of Monocyte-Derived Dendritic Cells (moDCs)

Mature moDCs were generated as per a previously published protocol [52]. A total of 1.5 × 10^6^ CD14+ monocytes were cultured in 2 mL X VIVO 15 medium (BioWhittaker, Lonza, Belgium) in six-well cell culture plates (Nunc, ThermoFisher Scientific, Waltham, MA, USA). GM-CSF 100 ng/mL (Peprotech, Cranbury, NJ, USA) and IL-4 100 ng/mL (Peprotech, Cranbury, NJ, USA) were added to the culture medium on day 0. After 24 h, maturation cytokines IL-1β 10 ng/mL, IL-6 10 ng/mL, TNF-α 50 ng/mL and PGE2 1 μM (Peprotech, Cranbury, NJ, USA) were added. Cells were harvested at 48 h.

### 4.4. Tolerogenic Stimuli

Dexamethasone (reconstituted in ethanol) 10^−7^ M (Peprotech, Cranbury, NJ, USA) was added, after 24 h of moDC culture, for a further 24 h to moDC cultures to induce a tolerogenic phenotype (Supplementary Figure S4).

### 4.5. MERTK Signalling

Recombinant human GAS6 (reconstituted in sterile water), 100 ng/mL (RnD Systems, Minneapolis, MN, USA), human protein S (reconstituted in sterile water), 5 μg/mL (Enzyme Research Laboratories, South Bend, IN, USA), UNC2025 (reconstituted in DMSO), 1 μM (Selleck Chemicals, Houston, TX, USA), MIPS15692 (reconstituted in DMSO), 10 μM and a specific MERTK inhibitor (Monash Institute of Institute of Pharmaceutical Sciences, Monash University, Parkville, Australia) were added to moDC cultures after 24 h of culture at the same time as dexamethasone for a further 24 h to assess effect on MERTK signalling (Supplementary Figure S4).

### 4.6. Lipopolysaccharide Treatment

At day 2 after generation of mature moDCs as per Section 4.3, cells were washed with PBS and fresh media was added containing either lipopolysaccharide, (Sigma, Darmstadt, Germany) 100 ng/mL, or no additional treatment for a further 24 h (Supplementary Figure S4).

### 4.7. Flow Cytometry

Phenotyping of primary human monocytes and monocyte-derived DCs was performed using flow cytometry. 1.5 × 10^6^ CD14+ monocytes were used for moDC differentiation in six-well cell culture plates (Nunc, ThermoFisher Scientific, Waltham, MA, USA). Differentiated cells were gently detached using PBS (without calcium and magnesium) and centrifuged at 300× *g* for 10 min at 4 °C to form a cell pellet. This was resuspended in 1 µL Fc receptor blocking antibody (Miltenyi Biotec, Bergisch Gladbach, Germany) and 49 µL FACS buffer per 10^6^ cells for 10 min at 4 °C. Cells were washed with FACS buffer and incubated for 30 min at 4 °C with fluorophore-conjugated antibodies or control isotype antibodies (Table 1). After incubation, cells were washed with FACS buffer to remove unbound antibodies and resuspended in an appropriate volume of FACS buffer for flow cytometry analysis, which was performed using CytoFlex LX (Beckman Coulter, Pasadena, CA, USA). Live cells were gated based on live–dead staining using 7-amino-actinomycin D (7AAD). Singlets were selected using forward light scatter (FSC) area and height. At least 10^4^ cells of interest were recorded and gated based on FSC and side light scatter (SSC) areas. Analysis was performed using FlowJo version 10.7.1.

### 4.8. Myelin Debris Efferocytosis Assay

Human myelin debris was fluorescently tagged with pHrodo iFL Green STEP Ester (P36012, ThermoFisher Scientific, Waltham, MA, USA). Then, 100 mg/mL aliquot of thawed human myelin was centrifuged at 14,800× *g* for 10 min at 4 °C. The supernatant was removed, and the pellet incubated in 200 μL 2 mM pHrodo solution in DMSO for 1 h at room temperature, protected from light. The fluorescently tagged myelin was washed with PBS twice, resuspended in PBS to 100 mg/mL and added to moDCs cultured from 1.5 × 10^6^ CD14+ monocytes in six-well cell culture plates (Nunc, ThermoFisher Scientific, Waltham, MA, USA) to achieve a final concentration of 1 mg/mL. Cell cultures were incubated for 1 h at 37 °C to allow phagocytosis of myelin debris. Cells were washed twice in warm PBS to remove excess cells and debris and harvested by mechanical dissociation before proceeding for analysis by flow cytometry.

### 4.9. Cytokine and Chemokine Analysis

Cytokine concentration quantification in cell culture supernatant (either fresh or stored at −20 °C) was performed using the Human Essential Immune Response Panel LEGENDplex bead-based immunoassay (BioLegend, San Diego, CA, USA) according to manufacturer protocol. Samples were analysed using CytoFlex S (Beckman Coulter, Pasadena, CA, USA) flow cytometer, and data analysis was performed using the LEGENDplex™ Data Analysis Software Suite (Version 2023-02-15).

### 4.10. CD4+ T Cell Isolation and Proliferation Assay

Isolation of untouched CD4+ T-cells from PBMCs was performed using the CD4+ T Cell Isolation Kit, MACS MS or LS Separator column and QuadroMACS Separator (all Miltenyi Biotec, Bergisch Gladbach, Germany) according to manufacturer protocol.

CellTrace™ Violet (CTV) proliferation kit (ThermoFisher Scientific, Waltham, MA, USA) was used to assess proliferation of magnetically isolated CD4+ T cells according to manufacturer protocol. Isolated cells suspended in PBS at 10^6^ cells/mL were incubated for 20 min at room temperature, protected from light with CellTrace™ stock solution and diluted in DMSO immediately prior to use. Then, 1 µL of CellTrace™ stock solution in DMSO was added per 1 mL of cell suspension in PBS. Culture medium of 5 times the original staining volume was added to the cells and incubated for a further 5 min. Cells were centrifuged at 300× *g* for 10 min to obtain a cell pellet and resuspended in warmed culture medium. Then, 10^5^ cells were analysed by flow cytometry immediately after staining for unstimulated control and to assess the purity of isolated CD4+ T-cells.

CTV-labelled CD4+ T-cells were co-cultured with moDCs at a ratio of 2:1 (3 × 10^6^ CD4+ T cells added to moDCs differentiated from 1.5 × 10^6^ CD14+ monocytes) for 7 days at 37 °C in 5% CO_2_. Cells were harvested for FACS analysis with unstained cells used as negative unstained control and unstimulated cells immediately after CTV labelling as positive control. Discrete peaks on the CTV fluorescence histogram were counted as successive generations of CD4+ T-cells.

### 4.11. Western Blot

We used 5 × 10^6^ CD14+ monocytes for moDC differentiation in 60 mm cell culture dishes (Nunc, ThermoFisher Scientific, Waltham, MA, USA) for Western blot experiments. Cultured cells were harvested by manual aspiration using chilled PBS and lysed in 10 mM Tris pH 8, 150 mM NaCl, 1 mM EDTA pH 8, Igepal 1% and protease/phosphatase inhibitors. Protein determination was performed using the Bradford reagent method. Then, 30 μg total protein was loaded per lane on a 4–12% gel (Invitrogen, Waltham, MA, USA) and run under reducing conditions in MOPS running buffer at 200 V for approximately 30 min. Transfer to PVDF membrane was performed using Bio-Rad Transblot Turbo mini or midi gels (Bio-Rad, Hercules, CA, USA). The membrane was washed in TBST (10 mM Tris pH 8, 150 mM NaCl, 1 mM EDTA pH 8, 0.1% Tween), blocked in 5% milk powder for 15 min at room temperature, then incubated with the primary antibody (MERTK, Abcam, Boston, MA, USA or phospho-MERTK, FabGennix, Frisco, TX, USA) diluted 1:1000 in TBST containing 2% BSA and 0.03% sodium azide overnight at 4 °C. The following day, the membrane was rinsed in TBST followed by incubation with the secondary antibody (HRP-linked anti-rabbit antibody, Cell Signalling Technology, Danvers, MA, USA) and diluted 1:3000 in TBST for 1 h at room temperature. The membrane was washed in TBST before visualising using chemiluminescent substrate. Imaging was performed using the ChemiDoc MP imaging system (Bio-Rad, CA, USA). Densitometry of MERTK and pMERTK bands was quantified relative to ERK expression.

### 4.12. Apoptotic Jurkat Cells Efferocytosis Assay and Immunofluorescence

#### 4.12.1. Jurkat Cell Culture and Apoptosis Induction

Jurkat cells were cultured in suspension in RPMI 1640 + 10% FBS at 37 °C in 5% CO_2_, and maintained by passaging 1:5 into fresh, pre-warmed media every 3–5 days. Apoptotic cells were prepared by allowing the Jurkat culture to grow to high density (4–5 days after passaging). Cells were pelleted by centrifuging at 500× *g* for 5 min, then resuspended in 1 mL of serum-free RPMI 1640 medium containing 1 μM staurosporine (Selleck Chemicals, Houston, TX, USA). Cells were incubated for 16 h at 37 °C in 5% CO_2_ to induce apoptosis. Apoptosis of cells was confirmed by staining with annexin V and 7-AAD (Biolegend, San Diego, CA, USA), as per the manufacturer’s protocol, and FACS analysis to confirm presence of annexin V-positive cells.

#### 4.12.2. Jurkat Cell Labelling

Cell Tracker Green CMFDA dye (ThermoFisher Scientific, Waltham, MA, USA) was used to fluorescently label apoptotic Jurkat cells. A working dye solution of 10 mM was prepared by dissolving the lyophilised product in DMSO. The working stock was diluted to 5 μM working concentration in serum-free RPMI 1640 medium and warmed to 37 °C. Apoptotic Jurkat cells were pelleted by centrifugation and incubated in the Cell Tracker Green working dye solution for 30 min at 37 °C. Stained cells were centrifuged to remove the working dye solution and resuspended in RPMI 1640 + 10% FBS.

#### 4.12.3. Efferocytosis of Apoptotic Jurkat Cells by moDCs

Apoptotic stained Jurkat cells, prepared as above, were pelleted by centrifuging at 500× *g* for 5 min and resuspended in RPMI 1640 + 10% FBS and 100 μL per well of moDCs. An apoptotic target/efferocyte ratio of 3:1 was used. The suspension containing 7.5 × 10^5^ apoptotic cells was added dropwise to 2.5 × 10^5^ moDCs cultured on glass coverslips in 24-well tissue culture plates (Nunc, ThermoFisher Scientific, Waltham, MA, USA). The culture plate was centrifuged at 200× *g* for 1 min to force contact between moDCs and Jurkat cells. The plate was incubated at 37 °C in 5% CO_2_ for 60 min. Cells were then washed twice with 1 mL of room-temperature PBS to stop efferocytosis and remove non-efferocytosed apoptotic cells.

### 4.13. Immunocytochemistry

Following efferocytosis, cells were fixed by adding 500 μL per well of 4% PFA for 10 min at room temperature. Cells were washed 3 times with PBS (with calcium and magnesium), 500 μL per well, and blocked with blocking buffer (0.3% Triton, 10% goat serum (Merck KGaA, Darmstadt, Germany) in PBS), 500 μL per well, for 1 h at room temperature. Cells were washed 3 times with PBS (with calcium and magnesium), 500 μL per well, and incubated with anti-MHC-II antibody (sc-53896, Santa Cruz Biotechnology, Dallas, TX, USA) (1:200), 150 μL per well, overnight at 4 °C. Cells were washed 3 times with PBS (with calcium and magnesium), 500 μL per well, and incubated with secondary antibody goat anti-mouse Alexa 647 (115-605-146, Jackson Immunoresearch, West Grove, PA, USA) (1:200), 150 μL per well, for 45 min at room temperature. Cells were washed 3 times with PBS (with calcium and magnesium), 500 μL per well. Cells were then incubated with anti-Rab17 antibody (17501-1-AP, Proteintech, Rosemont, IL, USA) (1:200), 150 μL per well, overnight at 4 °C. Cells were washed 3 times with PBS (with calcium and magnesium), 500 μL per well, and incubated with secondary antibody goat anti-rabbit Alexa 594 (111-585-003, Jackson Immunoresearch, PA, USA) (1:200) and Hoescht (Invitrogen, Waltham, MA, USA) (1:10,000), 150 μL per well, for 45 min at room temperature. Cells were washed 3 times with PBS (with calcium and magnesium), 500 μL per well, and coverslips mounted on slides using a drop of MOWIOL.

### 4.14. Image Analysis

Stained cells on coverslips were imaged using a Leica SP8 confocal microscope and Leica LAS X software version 5.1.0. Z-stack images of cells were captured with at least 60 cells per condition in three regions. Image analysis and cell counting were performed in ImageJ software version 1.53t. To quantify proportions of cells undergoing efferocytosis in each condition, the number of moDCs (nuclei/blue) bound to apoptotic Jurkat cells (green) was divided by total numbers of cells (nuclei/blue). Proportions of cells undergoing efferocytosis and not undergoing efferocytosis that expressed Rab17, MHC-II or both, respectively, were then quantified.

### 4.15. Statistical Analysis

Statistical analyses were performed using GraphPad Prism v9 (GraphPad Software, San Diego, CA, USA). The Shapiro–Wilk test for normality was performed. For normally distributed data, *t*-test or one-way ANOVA were used to compare means between groups followed by Fisher’s Least Significant Difference for pre-selected conditions indicated by comparisons shown in each figure. Where the normality test was not passed, a Kruskal–Wallis test was used to compare means between groups followed by Dunn’s post-hoc test for pre-selected conditions indicated by comparisons shown in each figure. Normally distributed data are expressed as mean ± standard deviation (SD). Non-parametric data are expressed as median ± interquartile range (IQR). A *p*-value of <0.05 was considered statistically significant.

## Figures and Tables

**Table 1:** Fluorophore-conjugated antibodies and dilutions used for flow cytometry experiments.

Reagent	Dilution	Source	Catalogue Number
Anti-human MERTK APC-conjugated IgG2b antibody	1:10	R&D Systems, Minneapolis, MN, USA	FAB8912A
Anti-mouse APC-conjugated IgG2b isotype control antibody	1:10	R&D Systems, USA	IC0041A
Anti-human HLA-DR (clone L243) APC Cyanine 7-conjugated antibody	1:100	BD Bioscience, Franklin Lakes, NJ, USA	641393
Anti-human CD40 (clone HB14) APC Cyanine 7-conjugated antibody	1:10	BioLegend, San Diego, CA, USA	313018
Anti-human CD80 (clone 2D10) Brilliant Violet 421-conjugated antibody	1:10	BioLegend, USA	305222
Anti-mouse Brilliant Violet 421-conjugated IgG1κ isotype control antibody	1:10	BioLegend, USA	400158
Anti-human CD83 (clone HB15e) Alexa Fluor 488-conjugated antibody	1:100	BioLegend, USA	305314
Anti-mouse Alexa Fluor 488-conjugated IgG1κ isotype control antibody	1:100	BioLegend, USA	400132
Anti-human CD86 (clone BU63) Brilliant Violet 605-conjugated antibody	1:50	BioLegend, USA	374214
Anti-mouse Brilliant Violet 605-conjugated IgG1κ isotype control antibody	1:50	BioLegend, USA	400162
Anti-human DC-SIGN (clone 9E9A8) PE-conjugated antibody	1:250	BioLegend, USA	330105
Anti-mouse PE-conjugated IgG2b isotype control antibody	1:250	BioLegend, USA	400213
Anti-human CD4 (clone VIT4) PerCP-conjugated antibody	1:50	BioLegend, USA	130-113-217

## Data Availability

Data sharing not applicable.

## Supplementary Materials

The following supporting information can be downloaded at: https://www.mdpi.com/article/10.3390/ijms242115903/s1.
