# Supplementary material for: The Tolerogenic Influence of Dexamethasone on Dendritic Cells Is Accompanied by the Induction of Efferocytosis, Promoted by MERTK
Source: Int J Mol Sci. 2023 Nov 2;24(21):15903. doi: 10.3390/ijms242115903 (PMC10650502; doi:10.3390/ijms242115903)
Supplement: Supplementary file 1 [file ijms-24-15903-s001.zip › ijms-2662327-supplementary.pdf]

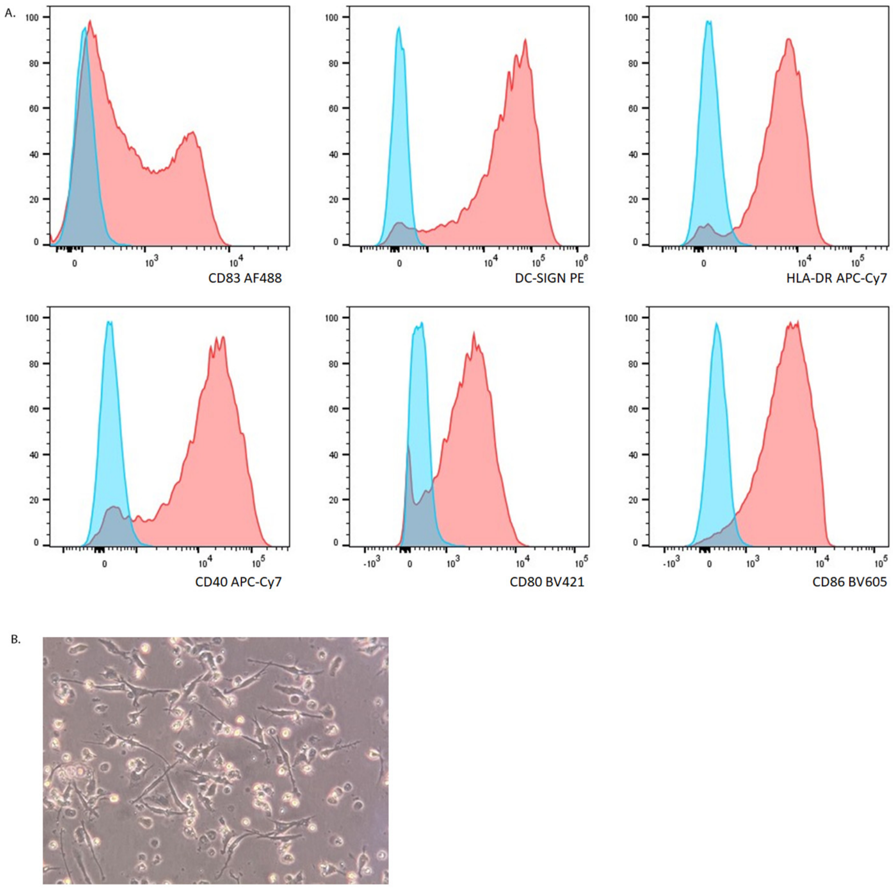

Figure S1. Representative FACS histograms showing monocyte-derived DCs express characteristic cell surface markers CD83, DC-SIGN, HLA-DR, CD40, CD80 and CD86 (blue represents isotype control, red represents indicated marker) (A) and have appropriate morphology on light microscopy (cells with cytoplasmic projections (dendrites) (B).

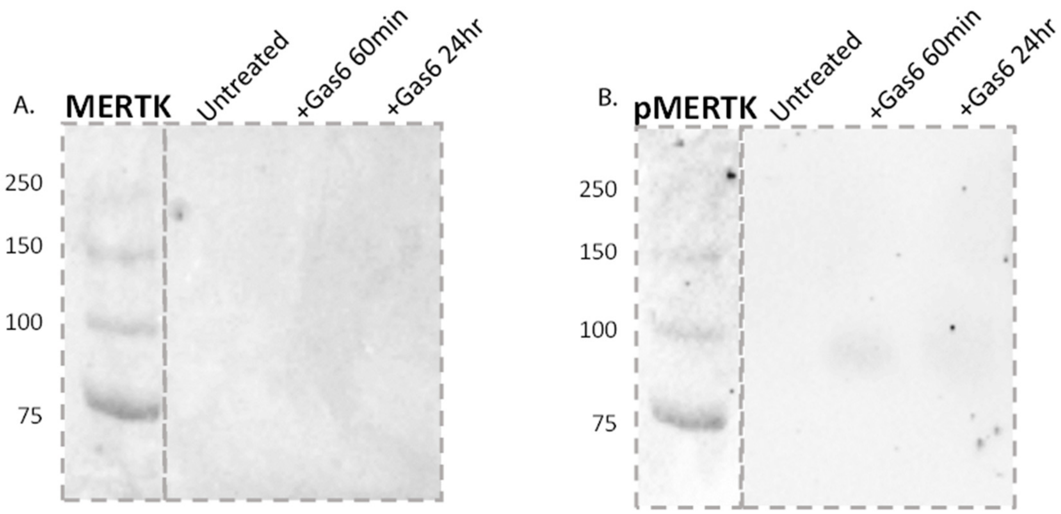

Figure S2. moDCs treated with GAS6 only for 60 minutes or 24 hours did not demonstrate MERTK expression (A) or phosphorylation (B) on Western blot. Grey dotted lines indicate different blots that have been spliced together to remove extraneous lanes. Level of MERTK expression and phosphorylation were quantified using densitometry.

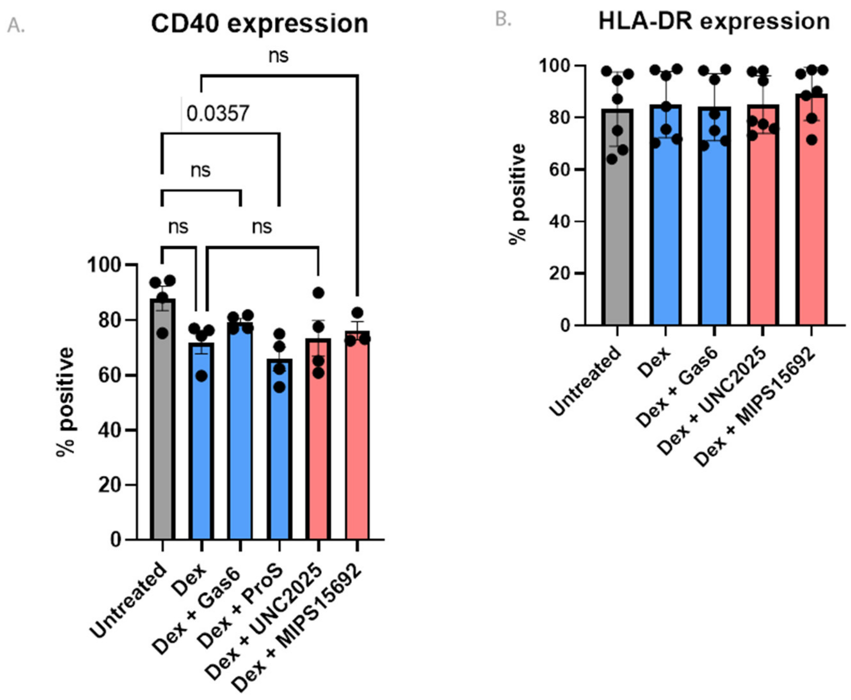

Figure S3. Dexamethasone 10^{-7}M treatment for 24 hours led to a lower proportion of moDCs expressing co-stimulatory molecule CD40 (n=4) but the difference was not statistically significant. UNC2025 1μM and MIPS15692 10μM for 24 hours did not have any additional effect on expression levels. One-way ANOVA was used to compare means between groups followed by Fisher's Least Significant Difference for pre-selected conditions indicated by comparisons shown in the figure (A). There were no significant differences in proportions of moDCs expressing cell surface HLA-DR in all treatment conditions (n=7) (B).

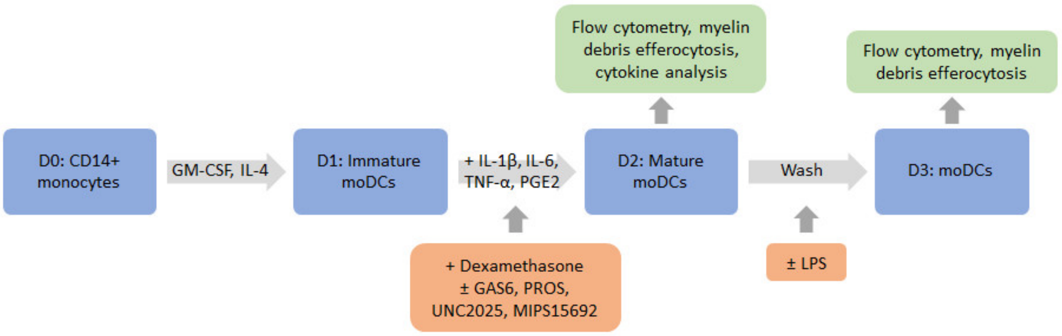

Figure S4. A schema depicting the treatment protocol.
